# New Appliances and Things Medical

**Published:** 1898-12-24

**Authors:** 


### NEW APPLIANCES AND THINGS MEDICAL.

[We shall be glad to receive, at our Office, 28 & 29, Southampton Street, Strand, London, W.C., from the manufacturers, specimens of all new preparations and appliances which may be brought out from time to time.]

### LISTER'S DOUBLE CYANIDE GAUZE.

(GALEN MANUFACTURING COMPANY, LIMITED, LADYWELL, LONDON, S.E.)

THE double cyanide of mercury and zinc with which this gauze is impregnated has been found, after extensive surgical use, to fulfil the conditions anticipated by Lord Lister when he introduced it as a substitute for the ordinary sal-alembroth dressing. That is to say, it is a reliable antiseptic, and it is less irritating to the skin. The sample before us is manufactured by the well-known Galen Manufacturing Company, and may be relied upon as being prepared according to the method approved of by Lord Lister.

### BEEF SUET.

(THOMAS DICKSON AND SON, LTD., EDINBURGH.)

THE beef suet which is specially prepared by the above firm is sent out in two forms, either in cakes or granulated. It will keep a considerable while, owing to the fact that it is free from impurity, carefully refined, and possesses a low degree of moisture. Experience has shown that prepared suets of this kind are economical in use; they save an immense amount of time in preparation and mincing, and, with all the care in the world, the result from the hands of the cook compares most unfavourably with that which is manufactured on a large scale with modern appliances specially devised for the purpose. Now that Christmas is close upon us, with its heavy demand for beef suet, it may be of value to housekeepers to know that, as far as purity and economy are concerned, there is no better family suet than that of Thomas Dickson and Son.

### PEPTOGENIC MILK POWDERS (FAIRCHILD).

(BURROUGHS AND WELLCOME, SNOW HILL BUILDINGS, E.C.)

BY means of the above peptogenic powders it is said that ordinary cows' milk may be readily, and at a moment's notice, modified to the standard of human milk. The directions with each bottle admit of no possible misunderstanding on the part of the nurse. The method has already been largely adopted, and in the majority of cases with complete success. Where it has failed the cause was probably to be sought in conditions outside the control of the nurse. We would point out, however, that there is no one standard for mother's milk, and hence there can be none for a milk that is modified to replace it. The percentage composition of human milk varies according to the stage of lactation, and especially so in that which is first secreted—and it is at this stage that children who are artificially fed first sow the seeds of intestinal trouble, a trouble which may prove eventually fatal. Another point we would draw attention to is that it must be remembered that by this method the milk is partially digested—not so completely, it is true, as is the case with the ordinary artificially prepared infants' foods, but sufficiently digested to shield the natural digestive powers of the gastro-intestinal tract from what should be its normal functions. This may lead to inadequate activity at a later date.

### PEPTONUM CARNIS.

(LIEBIG'S EXTRACT OF MEAT COMPANY.)

THIS preparation contains a maximum of nutriment in a minimum of space, and being in a digested state ready for absorption it constitutes, from a physiological standpoint, an absolutely economical food for invalids and debilitated persons. The albumoses, peptones, and extractives, of which it mainly consists, are not only foods from which there is no waste, but they are also stimulants to the system, as is very readily demonstrated by the improvement in the pulse after administration.

### WARD'S LIQUEUR SCOTCH WHISKY.

(15, MAITLAND STREET, EDINBURGH.)

THIS whisky is specially designed for the use of invalids. It appears to be a spirit of good quality, mellow, and of pleasant flavour, and well suited for the purposes of the sick-room.

